# Shifting mammal communities and declining species richness along an elevational gradient on Mount Kenya

**DOI:** 10.1002/ece3.11151

**Published:** 2024-04-09

**Authors:** Matthew H. Snider, Kristofer M. Helgen, Hillary S. Young, Bernard Agwanda, Stephanie Schuttler, Georgia C. Titcomb, Douglas Branch, René Dommain, Roland Kays

**Affiliations:** ^1^ Department of Forestry and Environmental Resources North Carolina State University Raleigh North Carolina USA; ^2^ Australian Museum Research Institute Sydney New South Wales Australia; ^3^ Department of Ecology, Evolution and Marine Biology University of California Santa Barbara Santa Barbara California USA; ^4^ Mammalogy Section National Museums of Kenya Nairobi Kenya; ^5^ North Carolina Museum of Natural Sciences Raleigh North Carolina USA; ^6^ Department of Fish, Wildlife, and Conservation Biology Colorado State University Fort Collins Colorado USA; ^7^ Department of Applied Sciences University of the West of England Bristol UK; ^8^ Earth Observatory of Singapore Nanyang Technological University Singapore Singapore; ^9^ National Museum of Natural History Smithsonian Institution Washington DC USA

**Keywords:** beta‐diversity, energy richness hypothesis, habitat specialization, mid‐domain effect, relative abundance, species richness

## Abstract

Conservation areas encompassing elevation gradients are biodiversity hotspots because they contain a wide range of habitat types in a relatively small space. Studies of biodiversity patterns along elevation gradients, mostly on small mammal or bird species, have documented a peak in diversity at mid elevations. Here, we report on a field study of medium and large mammals to examine the impact of elevation, habitat type, and gross primary productivity on community structure. Species richness was observed using a camera trap transect with 219 sites situated across different habitat types from 2329 to 4657 m above the sea level on the western slope of Mt Kenya, the second highest mountain in Africa. We found that the lowest elevation natural habitats had the highest species richness and relative abundance and that both metrics decreased steadily as elevation increased, paralleling changes in gross primary productivity, and supporting the energy richness hypothesis. We found no evidence for the mid‐domain effect on species diversity. The lowest elevation degraded Agro‐Forestry lands adjacent to the National Park had high activity of domestic animals and reduced diversity and abundance of native species. The biggest difference in community structure was between protected and unprotected areas, followed by more subtle stepwise differences between habitats at different elevations. Large carnivore species remained relatively consistent but dominant herbivore species shifted along the elevation gradient. There was some habitat specialization and turnover in species, such that the elevation gradient predicts a high diversity of species, demonstrating the high conservation return for protecting mountain ecosystems for biodiversity conservation.

## Graphical abstract

Our study intensively surveyed the western slope of Mt Kenya using a camera trap array covering a 2300 m elevation gradient with 219 sites. We found that relative abundance and species richness decreased as gross primary productivity deceased and elevation increased.

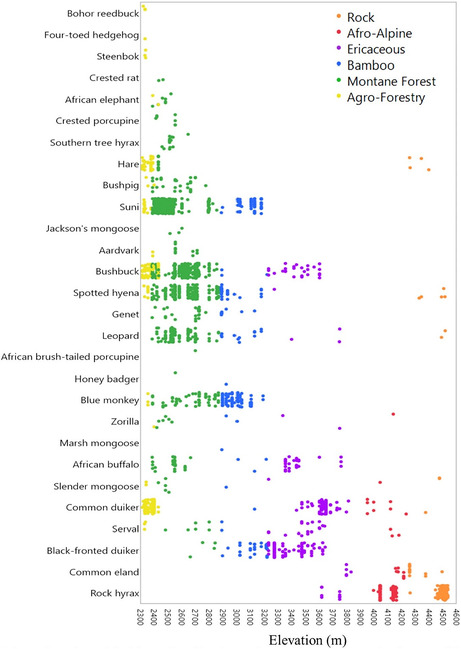

## 1 INTRODUCTION

Mountains are critically important for conservation because they provide diverse habitats for a wide range of species (Dirnböck et al., 2011) and, especially in light of climate change, because they provide climate refugia for many species (McCain & Colwell, 2011). Temperature and rainfall patterns in mountains can change dramatically across a relatively short linear distance, creating varied habitats and microclimates, that in turn create a mosaic of niches for specialist species. Tropical montane environments in particular are essential hotspots for vertebrate diversity (Rahbek, Borregaard, Colwell, et al., 2019). However, mountain biodiversity is now at risk due to increasing temperatures (Pauchard et al., 2016), which especially threaten species confined to higher elevations. Upslope shifts in species ranges and vegetation zones can result in the loss of suitable (tropical‐alpine) habitats following extensive warming (Dirnböck et al., 2011). This phenomenon, referred to as the “escalator to extinction” has been detected in bird communities in tropical montane habitats (Marris, 2007; Sekercioglu et al., 2008). Thus, documenting the pattern of change of biodiversity along elevational gradients has important conservation applications (Rahbek, Borregaard, Antonelli, et al., 2019).

Understanding the drivers of mountain biodiversity may help to identify conservation measures that avoid “escalator to extinction” scenarios, or to predict how habitat loss will affect mountain communities. Various hypotheses have been proposed to explain the high diversity of mountainous regions. Some studies of biodiversity change along elevational gradients have found that middle elevation domains have the highest diversity (Colwell & Lees, 2000). The prevailing explanation for this pattern is that tolerance ranges for both low elevation and high elevation specialists converge at mid‐elevations resulting in representation from both of these groups in the same intermediary spaces, commonly referred to as the mid‐domain effect (Colwell & Lees, 2000). This pattern has been shown for a variety of smaller species including small mammals, birds, herpetofauna, and snails across a wide range of temperate and tropical mountains (Malonza, 2015; McCain, 2004; Pan et al., 2016; Patterson et al., 1998; Rickart et al., 2011; Tattersfield et al., 2001). There has been relatively little study of these patterns in medium and large mammals (>500 g, hereafter referred to as “large mammals” in line with other co‐regional studies (Gebert et al., 2019)), which might be expected to show different patterns of diversity due to their larger spatial and/or larger energy requirements. Since larger mammals range more widely and live at lower density, they might be less likely to survive as specialists on narrow bands of suitable habitat, as often seen in smaller species (Sanchez‐Cordero, 2001) although a relative lack of large mammal focused studies leave these patterns in question.

The energy richness hypothesis (Wright, 1983) provides an alternative prediction that high elevations should have both fewer individuals and lower diversity than lower elevations due to the lower levels of primary productivity associated with alpine plant communities. While this theory has typically been tested along latitudinal gradients, it could be even more appropriate for high elevation tropical habitats because the seasonal variation that confounds the latitudinal explanations is less pronounced in equatorial montane environments (Malhi et al., 2017). This hypothesis predicts that measures of primary productivity describe how much energy is available in a habitat to support animals across all trophic levels. One potential mechanism for this is described by the “more‐individuals hypothesis” (Storch et al., 2018), where higher energy availability supports more individuals, allowing more species to have viable population sizes. Thus, the energy richness hypothesis (and “more‐individuals mechanism”) predict that patterns of mammal relative abundance and diversity should follow the trend of productivity along elevational gradients, which generally decreases with increasing elevation. This differs from the mid‐domain effect in that lower elevations (and higher areas of productivity) may have lower mammal relative abundance and diversity than intermediate elevations (Rahbek, 1995).

Surprisingly, few studies have examined large mammals along an elevational gradient; the most similar investigation was conducted on Mt Kilimanjaro (Gebert et al., 2019). While this study detected a mid‐domain peak in richness at about 1800 m, a lack of relative abundance data still limits our knowledge of elevational patterns of diversity in larger mammals in contiguously protected landscapes. We also have little insight as to whether elevational patterns are determined by overlapping ranges of elevational specialists, as has been observed for smaller species, or by patterns of primary productivity, as seen across latitudinal gradients.

The Kenyan highlands are among the most species‐rich areas of equatorial Africa, harboring high levels of threatened and endemic biota. Indeed, this region is recognized as a global epicenter of threatened biodiversity, having been designated as an Eastern Afromontane Biodiversity Hotspot (Mittermeier et al., 2004). Significantly, a number of highly‐threatened Guineo‐Congolian lowland rainforest species occur isolated from their main ranges in montane habitats on Mt Kenya (Dommain et al., 2022) such as the bongo (*Tragelaphus eurycerus isaaci*), black‐fronted duiker (*Cephalophus nigrifrons hooki*) and potto (*Perodicticus ibeanus stockleyi*), (Butynski & De Jong, 2017; Kingdon et al., 2013). The Mt Kenya highlands are noted as an area of high priority for conservation investment and protection in Africa due to the potential to protect numerous species in a small area (Mittermeier et al., 2011). In terms of biodiversity patterning, several studies have investigated trends in small animal and plant species along elevational gradients. For example, Musila et al. (2019) recently showed that small mammal species diversity exhibited a mid‐domain effect on the east (wetter) side of Mt. Kenya but that there was little variation in species richness on the (drier) northwestern side of the mountain. In contrast, studies on mollusks on the mountain showed that diversity steadily decreased with elevation (Tattersfield et al., 2001), and that herpetofauna diversity peaked twice (Malonza, 2015). Here we report the first systematic study of large mammal communities on Mt Kenya, adding to previous small scale or anecdotal reports, such as U.S. President Theodore Roosevelt's elephant hunt (Roosevelt, 1910) and work in the Teleki Valley of the mountain (Young & Evans, 1993).

**FIGURE 1:**
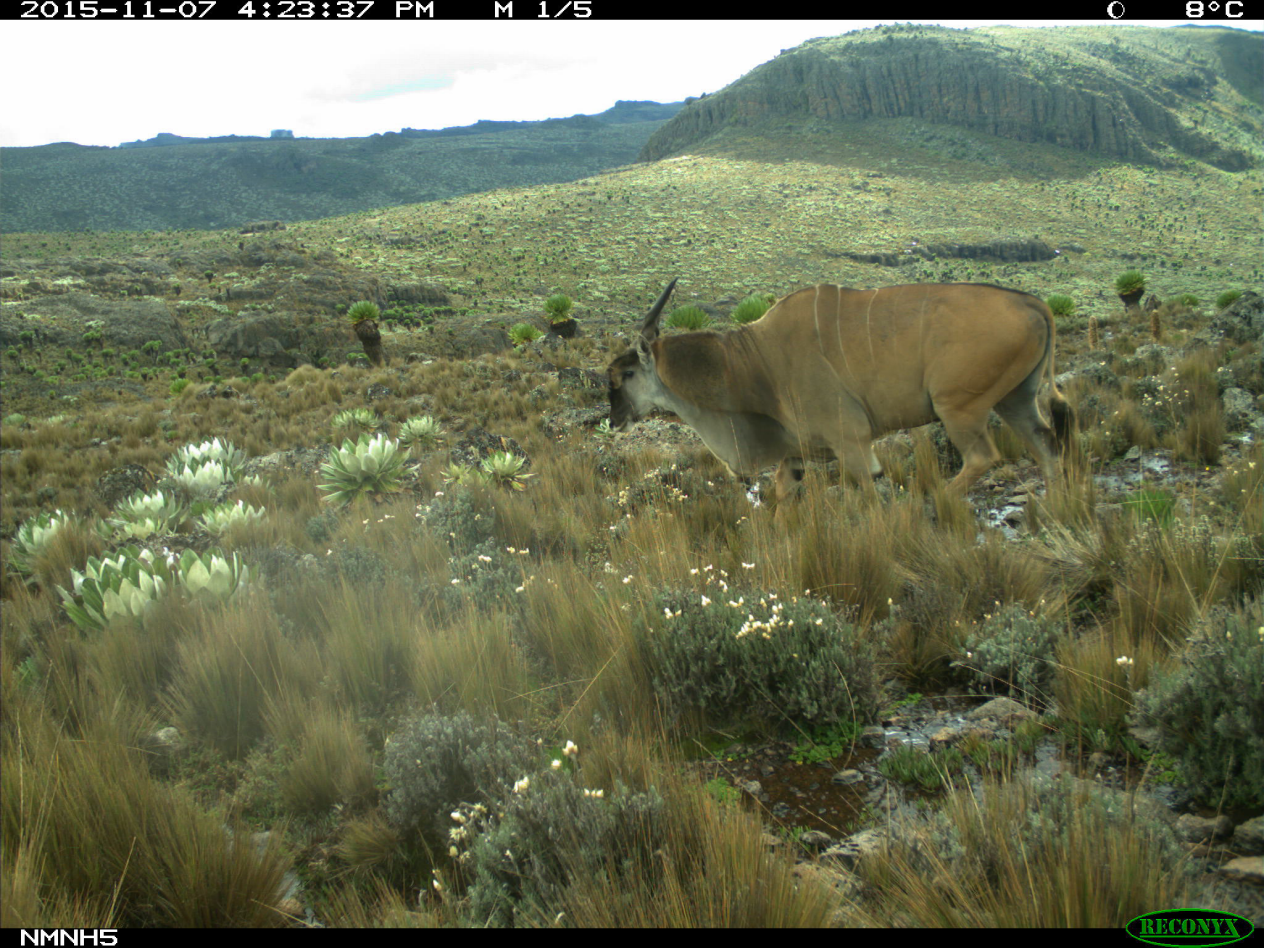
A male common eland (*Taurotragus oryx*) traversing the sparse vegetation of Mt Kenya's Afro‐alpine habitat at an elevation of 4164 m.

The goal of this paper is to test hypotheses about the determinants of diversity by analyzing changes in large mammal diversity along a 2328 m elevational gradient of Mt Kenya. We asked whether diversity is unimodal due to an overlap of ranges for the high elevation and low elevation specialists (resulting in a mid‐domain effect) or, alternatively, if diversity is negatively correlates with elevation as a result of resource limitations, as predicted by the energy richness hypothesis and many‐individuals mechanism. Because the natural landscapes of Mt Kenya are increasingly isolated and surrounded by land converted to rain‐fed cropland and non‐native forest plantations (including mixed agriculture small plot shamba farming, timber plantations, and livestock grazing) (Eckert et al., 2017), a secondary objective of our project is to establish which wildlife species can occupy disturbed habitat outside of protected areas. This study sheds light on elevational patterns in large mammal diversity, a largely missing component of elevational patterns across taxa, which tends to be dominated by work on smaller and less mobile taxa (Figure 1).

## 2 MATERIALS AND METHODS

### 2.1 Study site

**FIGURE 2:**
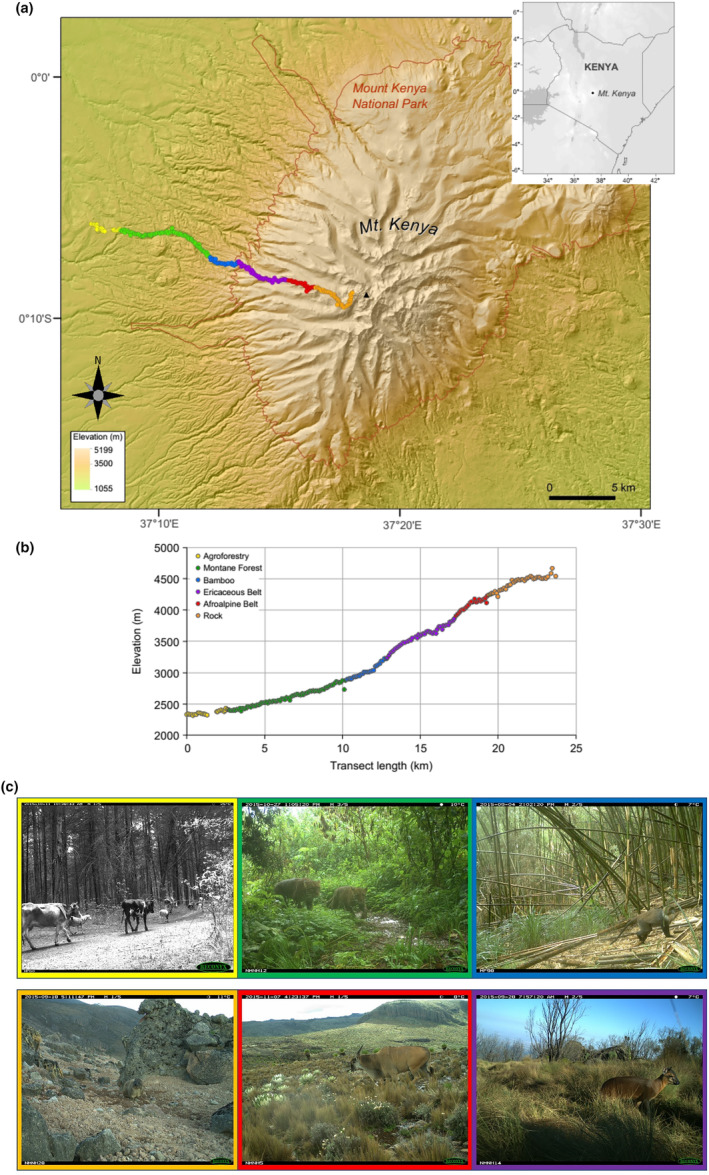
(a) Study area with transect of camera trap sites set every 100 m along the Burguret trail on the western slope of Mt. Kenya. Colored dots represent each camera trap location with the color denoting the habitat type (legend shown in b) (Map image: TanDEM‐X DEM ©DLR). (b) Camera trap sites shown by their elevation along the transect. Colored dots denote habitat type of each site. The legal park boundary is between the bamboo and ericaceous zones but the functional protected space extends down to the base of the Montane Forest (see the study site text for more details). (c) Examples of habitat types organized counter‐clockwise from the top left (lowest elevation) to bottom left (highest elevation) with corresponding color borders.

At an elevation of 5199 m above the sea level, Mount Kenya (00°9′ S, 37°19′ E) is the second‐highest peak in Africa and a truly equatorial mountain as its northern slopes straddle the equator (Figure 2a). Mt. Kenya is an extinct volcano formed over 5 million years ago which last erupted about 1 million years ago (Veldkamp et al., 2012).

The climate of the region exhibits strong rainfall seasonality but very small annual temperature fluctuations. The bi‐annual passage of the tropical rainbelt results in two distinct rain seasons between March and June and between October and December with intervening dry seasons (Nicholson, 2017). Due to a rain shadow effect, the northwestern side (and to a lesser extent, the western side investigated here) of Mt. Kenya is drier than the southeastern side, which receives moisture from the Indian Ocean (Baker, 1967; Coe, 1967; Downing et al., 2023; Smith & Young, 1987). From 1948 to 3000 m, rainfall increases and reaches an average of 1050 mm and a maximum of about 1600 mm per year on the western slope (study area) (Downing et al., 2023). Above this elevation, rainfall decrease to approximately 750 mm per year at the summit region (Baker, 1967; Coe, 1967). In contrast, temperatures continuously decrease with increasing elevations (Coe, 1967).

The climatic gradients are reflected in distinct vegetation zones, which comprise three main altitudinal belts: the Montane Forest belt, the Ericaceous belt, and the Alpine belt, typical for the highest mountains in East Africa (Allen, 1991; Coe, 1967; Hedberg, 1951; Smith & Young, 1987). Below an elevation of about 2200–2300 m the natural forest cover of Mt Kenya region has been almost completely lost and is replaced by agricultural fields and exotic timber plantations (*Cupressus lusitanica* and *Grevillea robusta* on the western side) (Kehlenbeck et al., 2011). The Montane Forest area on the mountain consists of three zones: montane rainforest (2400–2870 m), bamboo (2870–3220 m) and *Hagenia‐Hypericum* forest (3220–3285 m) (Table 1) (Hedberg, 1951). The Ericaceous belt stretches from 3285 to 3857 m and, on the western side, consists of a mosaic of shrublands dominated by tree heather (*Erica arborea)* and sedge‐grass swamps dominated by the sedge *Carex monostachya* and tossock grass *Festuca pilgeri*. The Afro‐Alpine belt (3857–4246 m) is characterized by tussock grasslands (*F. pilgeri*), dwarf shrubs (*Alchemilla agrophylla*), giant senecios (*Dendrosenecio keniensis*, *D. keniodendron*) and giant lobelias (*Lobelia telekii*, *L deckenii*). Above this is the windswept Rock zone (4246–4657 m) composed of craggy peak formations, diminishing glaciers, and rock fields with few scattered herbs, grasses, and mosses (Figure 2c).

**TABLE 1:** Habitat zones (Young & Evans, 1993) sampled with corresponding major vegetation, elevations, and number ~25‐day camera traps deployments during our study.

Habitat	Primary vegetation	Elevation range (m)	Number of cameras
Agro‐forestry	Exotic timber plantations (*Cupressus lusitanica*), small plot potato farming	2329–2401	21
Montane Forest	Mixed, old growth forest dominated by *Podocarpus latifolius*, *Juniperus procera*, understory partly by *Arundinaria alpina*	2401–2869	71
Bamboo zone	Monodominant stands of bamboo (*Arundinaria alpina*)	2869–3214	25
Ericaceous Belt	Combined zone of *Hagenia abyssinica* – *Hypericum revolutum* forest zone and Ericaceous belt (shrublands of *Erica arborea* and *Festuca pilgeri* tussocks, partly burned in 2012) and swamps of *Carex monostachya* and *F. pilgeri*)	3214–3857	43
Afro‐Alpine Belt	Stands of giant senecios (*Dendrosenecio* spp.) and giant lobelias (*Lobelia* spp.) among tussock grasslands dominated by *F. pilgeri* and dwarf shrubs of *Alchemilla agrophylla*	3857–4246	21
Rock	Bare ground and rocky outcrops with <10% vegetation cover	4246–4657	38

The greater Mt Kenya ecosystem features a border at 2401 m elevation that separates the Mt Kenya Forest Reserve, administered by the Kenya Forest Service (KFS), at lower elevations, and the greater Mt Kenya National Park management area (Figure 2a), administered by the Kenya Wildlife Service (KWS), at higher elevations. The Forest Reserve below 2401 m is a mixed‐use agricultural area that undergoes agricultural cycles where new timber saplings are planted after mature forest clearcut harvests, during which time the land is also used for small plot potato farming (called shambas) between the sapling rows by tenant farmers for the first 10 years. At intermediate timber growth stages, tenant farmers either interplant shade vegetables between the timber rows or move to the next timber clearcut plot and livestock is brought in to graze the intermediate to mature timber areas (Bussman, 1994; Eckert et al., 2017). In all stages this habitat type is under intensive land use and occupation, including the corresponding wildlife trapping/hunting. Above 2401 m, the National Park management area is strictly off limits to consumptive extraction or livestock use (Figure 2). Given that the conversion of natural habitat into agricultural habitat has rapidly increased over the last 40 years (Eckert et al., 2017), there is a sharp distinction between protected habitats at elevations above 2401 m in along the Burguret Trail and mostly disturbed habitat at lower elevations.

### 2.2 Data collection

We conducted this study along the western slope of Mt Kenya (00°8’S, 37°15’E) from August 29, 2015 through November 18, 2015. We positioned camera traps every 100 m along a 23.7 km long transect following the Burguret Trail (Figure 2) from elevations 2329 to 4657 m covering six main habitats (Table 1). Our camera trap transect followed routes used by the Smithsonian research teams accompanying former Roosevelt (1910) in order to consolidate logistics with concurrent Smithsonian small mammal research during our study period. To evaluate the importance of management difference systems, we also deployed cameras in the Agro‐Forestry plantation buffer zone at the boundary of the park to quantify wildlife species using this degraded habitat, and the use of the protected areas by neighboring livestock.

We deployed camera traps (Reconyx Hyperfire: RECONYX, Inc., Holman WI, USA) following best practices as evaluated in Kays et al. (2020), locked to trees and rocks at knee height (~50 cm) without bait (a configuration that enabled these camera traps to reliably detect mammals down to ~500 g), rotating to new sites within the same elevational range every 3–4 weeks, for a total of 239 trap locations across the six habitat types over a three‐month period. Sites were chosen using a stratified‐random design to cover off‐trail, game‐trail, and main‐trail locations with each camera spaced at least 100 m away from another camera in the same strata to prevent auto‐correlation (Kays et al., 2010, 2021). Cameras were set to take a series of 10 photos at 1 frame/s for each motion trigger, and to retrigger immediately if the animal was still in view. For analyses we combined consecutive triggers <60s apart into one “sequence” and counted the number of animals that passed through the field of view as a measure of group size. We stratified cameras along the main trail (68 cameras), game trails (75 cameras), and off trail (96 cameras).

We used the eMammal system to process the camera trap data by uploading each deployment, excluding false triggers, and recording each animal identified (McShea et al., 2016). Field staff initially identified animals detected before a second expert mammalogist team member reviewed identifications for taxonomic accuracy. For analysis, we used composite groups for three taxa (hares: *Lepus capensis*, *Lepus victoriae*; genets: *Genetta genetta*, *Genetta maculata*, and small rodents) that could not be consistently identified to species level in camera trap photographs (Table S1).

We related mammal species assemblages in specific elevational zones to the corresponding vegetation belts (Table 1). Vegetation belts and their elevational ranges were mapped along the Burguret Trail between August 19 and 31, 2015. Dominant plant species were identified with Agnew (2013) and Beentje (1994) and elevations recorded with handheld GPS (Garmin GPSmap 65s).

**FIGURE 3:**
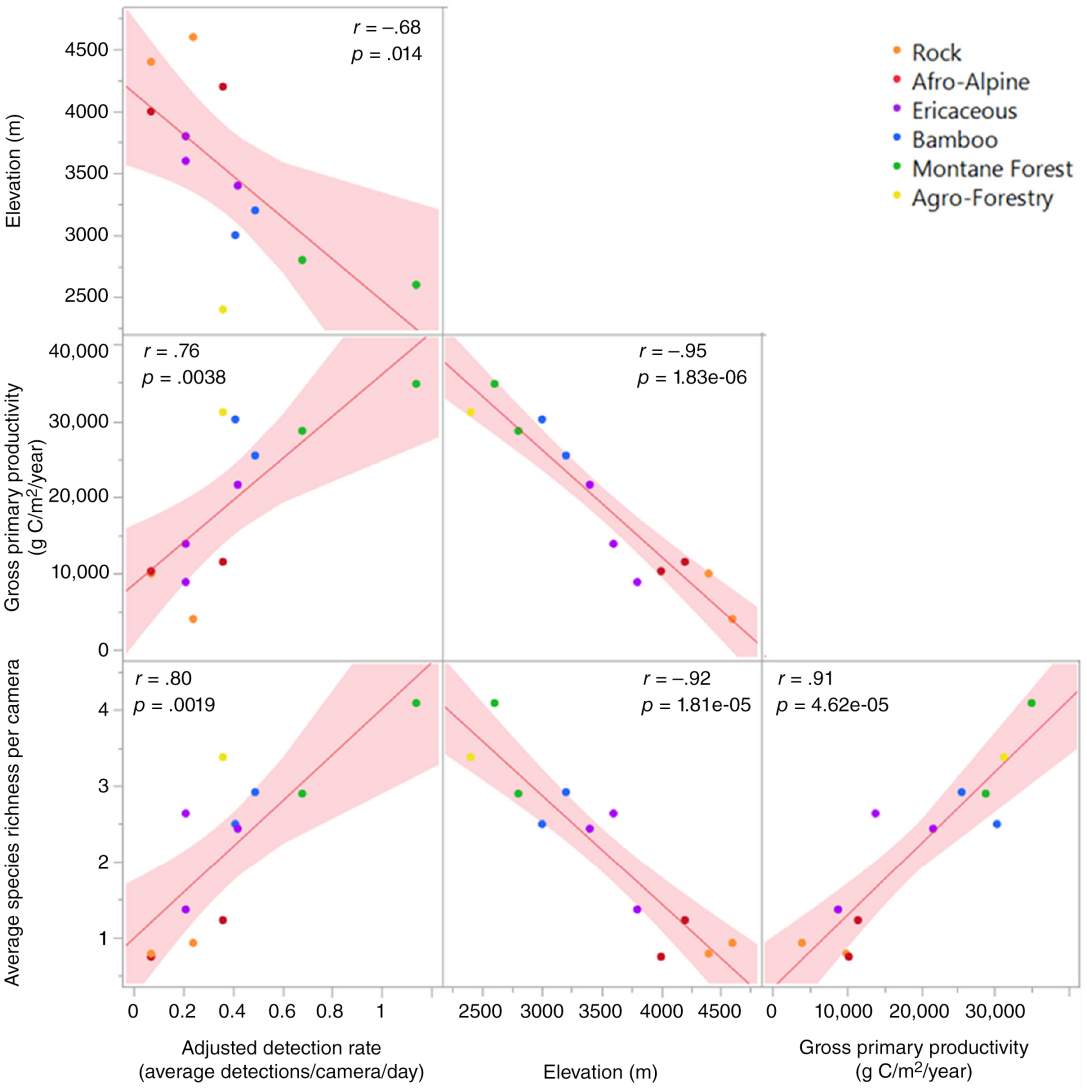
Correlation matrix between elevation, average species richness per camera, GPP, and adjusted detection rates. As elevation increased, the number of detected mammal species decreased. Each point represents the number of species detected, averaged across camera traps within 200 m elevation increments (cameras had lateral spacing of 100 m but varied in elevational spacing), with colors indicating habitat type. These 200 m elevation averages were tested for normality using the Shapiro–Wilk normality test (*p* = .29, normal distribution). As gross primary productivity increased, the average number of mammal species and average detection rate increased. As gross primary productivity increased the average rate of mammal detections per camera per night, an index of animal abundance, increased.

At each camera location, we calculated the gross primary productivity (GPP), a key indicator of ecosystem productivity. We used the NASA MODIS Terra data to estimate GPP with a spatial resolution of 1 km (Radeloff et al., 2019), which was the highest resolution available for our study period. Our transect of camera traps extended over 23.7 km, allowing us to observe and map variations on GPP across the elevational gradient (Figure 3). We used the software QGIS (QGIS Development Team, 2018) to assign each camera trap site a specific GPP value based on its location.

### 2.3 Analyses

To measure relative abundance for each species and camera deployment, we calculated the detection rate of each species as the sum of animals seen moving through the frame of view of the camera divided by the number of total trap nights. Previous studies using a variety of methods have shown that detection rate is closely correlated to animal density when camera deployments are unbiased (Nakashima, 2018; Rowcliffe et al., 2008). In addition to this raw rate, we also accounted for the fact that larger animals tend to trigger the camera from further distances than smaller animals by estimating detections/day/m^2^. We used the formula:

$$Detection\;correction\;=\;\frac{\text{Detection}\;\mathrm{rat}e^{-\mathrm{day}}}{1.65\times \text{Body mass}\;{\left(\mathrm{kg}\right)}^{\frac{1}{3}}}$$

from Rowcliffe et al. (2011) and obtained average body masses for all species from the Animal Diversity Web (University of Michigan Museum of Zoology, 2020). Due to the difficulty of sexing many of the species observed, we used the average body mass between male and female based on the Animal Diversity Web (University of Michigan Museum of Zoology, 2020). We used JMP software to calculate Pearson's correlation coefficient between the relative abundance and elevation, and also GPP and average species richness detected by cameras grouped by 200 m elevation change (Figure 3). These grouped values were tested for normality using the Shapiro–Wilk normality test. We used the *betapart* (Baselga & Orme, 2012) and vegan R packages (Oksanen et al., 2016) to calculate species richness and Shannon diversity, and to evaluate beta‐diversity between habitats based on the Sørenson similarity index. We used the function “estimateR” in *vegan* to calculate Chao species estimates to evaluate sample adequacy, in addition to generating a species accumulation curve. We then analyzed the relationship between diversity and elevation by fitting a Gaussian generalized linear model for Shannon diversity, and a Poisson generalized linear model for species richness data in R. We compared quadratic and linear fits using elevation and habitat zones as covariates of Shannon diversity and species richness of all mammal species by camera trap. We compared AICc values to determine which model best fit the data and tested for violations of model‐fitting assumptions using the DHARMa package (Hartig, 2020).

## 3 RESULTS

Of the 239 camera trap deployments, 219 were successful, (20 failures arose due to dead batteries, card errors, obstruction by falling vegetation, and hyena camera damage). Each deployment location was monitored for approximately 4 weeks before it was recovered and redeployed to the next target site. The successful deployments with at least 14 camera trap nights resulted in a total of 5756 trap nights in which 28 species of wildlife and four species of domestic livestock were detected, totaling 3018 wildlife observations and 2447 domestic species observations (see Table S1 for raw data and scientific names). Exact counts and detection rates per species can be found in Table S1.

### 3.1 Wildlife relative abundance

Within the national park there was a relatively consistent downward trend in wildlife abundance with increasing elevation when considering individual deployments (Figure S1), or averaging within habitat types (Figure S2). The relative abundance of all native species was negatively correlated with elevation (*r* = −.68, *p* = .01) and positively correlated with GPP (*r* = .91, *p* = 4.62e‐05, Figure 3). Outside the park, in the Agro‐Forestry zone, there was a high level of domestic animal activity and low abundance of native species (Figure S2).

### 3.2 Species richness and Shannon diversity

Our observations fell within Chao species richness estimates suggesting sampling adequacy over our research period (Figure S5). This was reinforced by our species accumulation curve that which leveled off at approximately 30 species over our 219 deployments (Figure S5). Native species richness declined steadily with elevation, and this effect was also explained by habitat type. Linear and quadratic models that included elevation and habitat performed similarly to models with only elevation (AICc range = 725.7–727.8) suggesting that adding habitat as a covariate only modestly improved model fit (Table 2), over models with elevation or habitat alone (Figure 3). Given that simpler models should be preferred in the case of similar competing models (Arnold, 2010), we concluded that the best model of species richness and Shannon diversity included only the elevation. We did not find significant evidence for heightened species richness or Shannon diversity at mid‐elevations, indicated by the lack of a significant negative second‐order polynomial coefficient in quadratic fits (richness *p* = .06, Shannon's diversity *p* = .55).

**TABLE 2:** Summary of generalized linear models of species richness (Poisson error structure) and Shannon diversity (Gaussian error structure) to elevation and habitat.

Covariates	AIC	dAICc	df
Species richness (Poisson models)			
Linear elevation + habitat	725.7	0	7
Quadratic (Elevation)	726.2	0.5	3
Quadratic (Elevation) + habitat	727.5	1.9	8
**Linear elevation**	**727.8**	**2.1**	**2**
Habitat	736.9	11.2	6
Shannon diversity (Gaussian models)			
**Linear elevation**	**247.8**	**0**	**3**
Quadratic (Elevation)	249.5	1.7	4
Linear elevation + habitat	252.3	4.5	8
Quadratic (Elevation) + habitat	254.2	6.4	9
Habitat	259.2	11.3	7

*Note*: The model containing only linear elevation (bold) was the most parsimonious model for both metrics of diversity, explaining a very similar degree of variation as other models that included habitat and quadratic terms, but using fewer degrees of freedom (Arnold, 2010).

### 3.3 Species‐specific patterns

**FIGURE 4:**
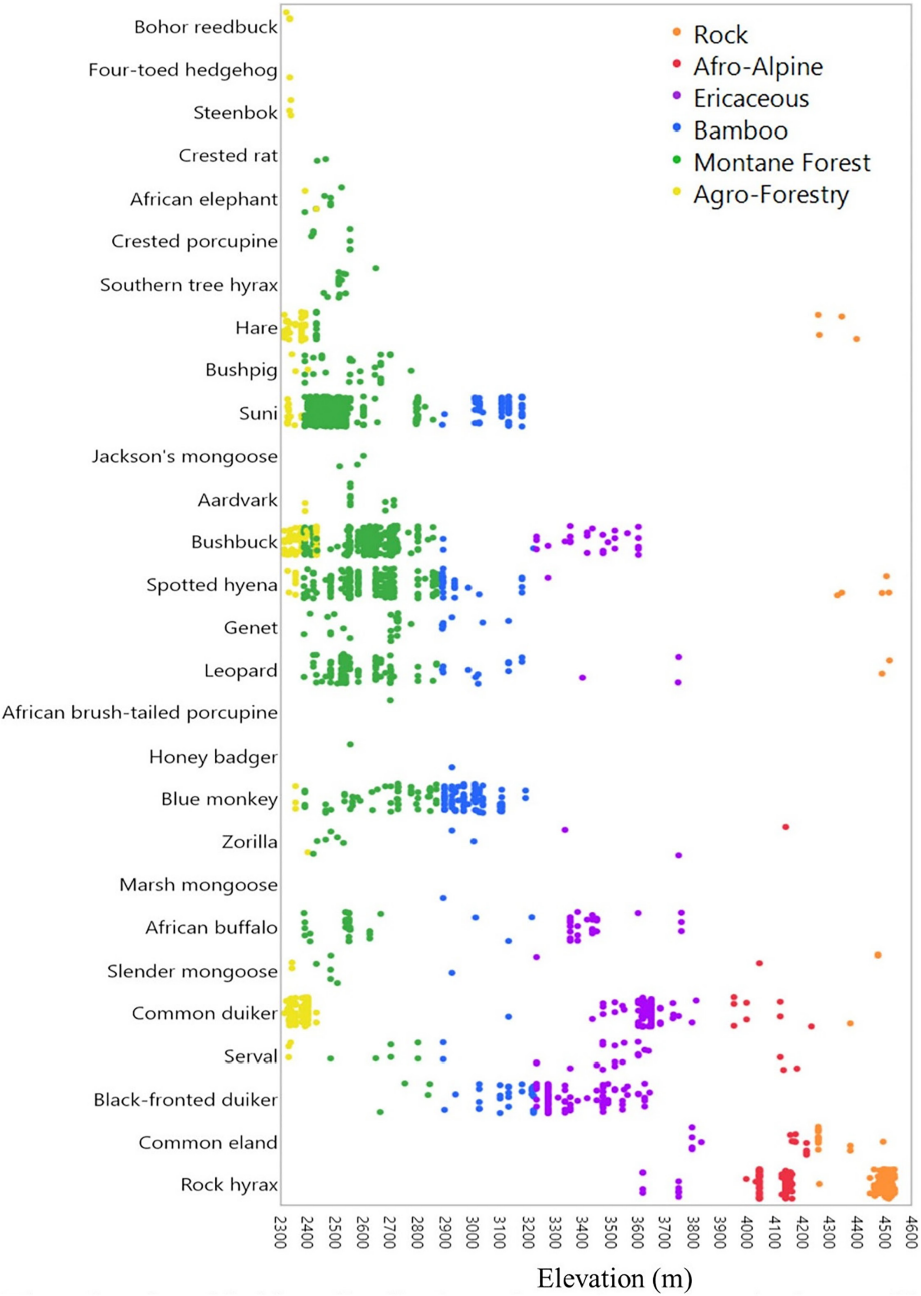
Elevational and habitat distributions for native mammals detected by 219 camera traps set along an elevational gradient on Mt Kenya. Each colored dot represents the detection of a species by a camera trap at that elevation ordered by each species mean detection elevation.

Changes in diversity across the elevational gradient were likely caused by different levels of habitat specificity across mammals (Figure 3). On one side of the spectrum were the strict habitat specialists, like the steenbok (*Raphicerus campestris*), that were found in only the Agro‐Forestry zone. Likewise, the crested porcupine (*Hystrix cristata*) and the southern tree hyrax (*Dendrohyrax arboreus*) were only detected in the Montane Forest of the National Park. Other species, like the suni (*Neotragus moschatus*), were commonly found in multiple zones, often occurring in high relative abundance in one zone and in moderate and low abundance in adjacent habitats (Figures S3 and S4). Interestingly, the common duiker (*Sylvicapra grimmia*) was found in high numbers in both the patchwork of Agro‐Forestry land and the high elevation Ericaceous belt but was only observed once in the denser Montane Forest or Bamboo zones that feel between these two zones, displaying a fidelity to habitats with similar vegetation characteristics. Finally, many carnivores (Carnivora), including leopard (*Panthera pardus*), spotted hyena (*Crocuta crocuta*), and zorilla (*Ictonyx striatus*) were habitat generalists that were detected across multiple habitat types and elevational zones (Figure 4).

Changes in species dominance across habitats and elevations helped us understand shifts in montane mammal communities on Mt Kenya (Figures S3 and S4). For herbivores, we found unique species combinations dominating each habitat. Specifically, suni and bushbuck (*Tragelaphus scriptus*) dominated in the dense vegetation of the lower tropical Montane Forests, blue monkeys (*Cercopithecus mitis*) and suni dominated in Bamboo, black‐fronted and common duiker dominated the Ericaceous zone, and the rock hyrax was by far the most common animal in the rocky Afro‐Alpine landscapes at the top of the mountain. Importantly, there are zones that, despite having lower than expected species richness, are critical habitats for certain species and which should be conserved. The pattern for carnivores was much different; apart from the serval (*Leptailurus serval*) being most common in the Ericaceous and Afro‐Alpine zones, leopard and spotted hyena were dominant carnivores across most habitats. Generalist carnivores, especially relatively large carnivores that are able to cover a wider range in search of food resources, appear to be more abundant across the mountain.

### 3.4 Community similarity

**FIGURE 5:**
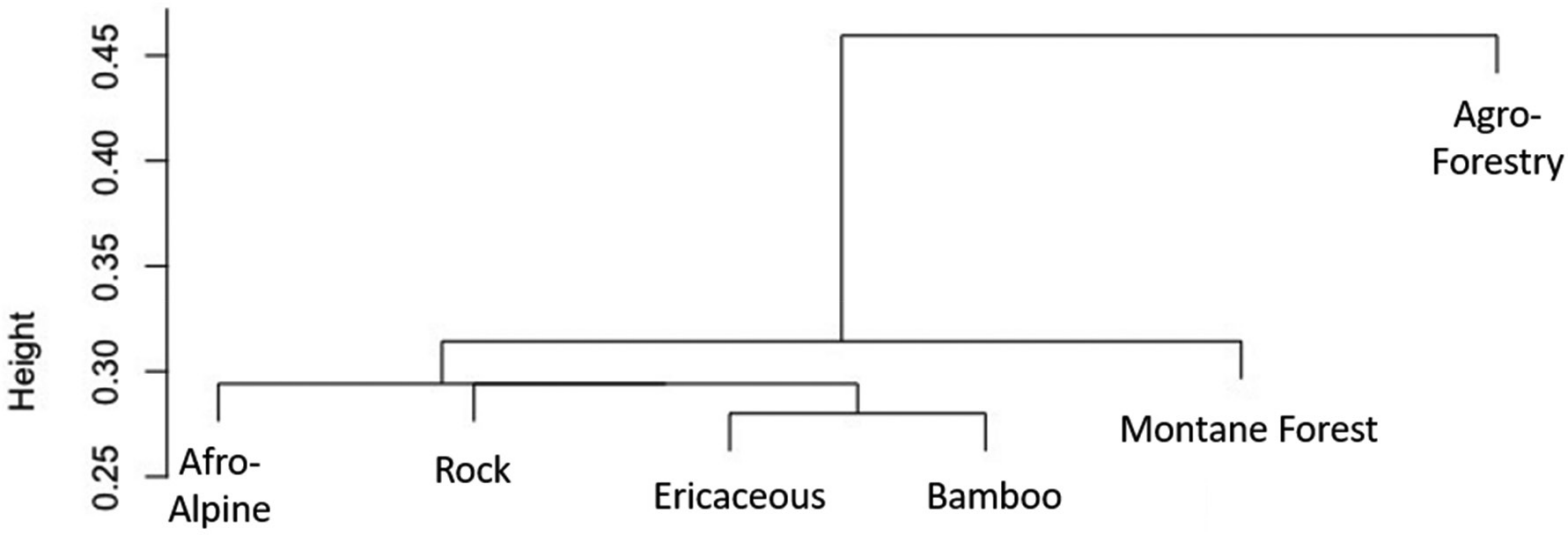
Sørenson similarity beta‐relateness index detailing similarities of species compositions of mammal detection by camera traps across six habitat types on Mt Kenya. Height denotes the similarity between mammal communities in that habitat types with shorter heights relative to each other being more closely related. Exact measures can be found in Table S2.

**FIGURE 6:**
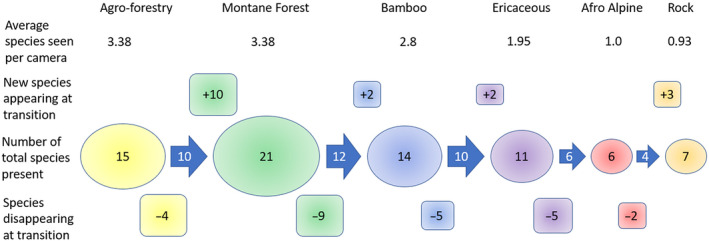
Mean species richness detected per camera and change in the diversity of mammals in major vegetation zones along an elevational gradient on Mt Kenya. This alpha diversity pattern resulted from a combination of species maintaining a presence from one adjacent habitat type to the next (arrows), species disappearing (lower boxes) or appearing (upper boxes) at each habitat transition.

Our Sørenson similarity index calculations of wildlife community beta‐diversity show that the wildlife species composition in the agro‐forestry is the most dissimilar from the undisturbed habitats, with various non‐montane species that were only recorded there (Figure 5, exact values in Table S2), such as the four‐toed hedgehog (*Atelerix albiventris*) and steenbok. Subdivided by habitat, alpha‐diversity changed from a maximum of 20 species in the more productive Montane Forest to a minimum of 6 species in the low productivity Afro‐Alpine and Rock habitats, centered around high elevation specialists like eland (*Taurotragus oryx*) and rock hyrax (Figure 6). This subdivision of habitat also mirrored patterns of elevation where higher elevation habitats had lower mean species richness by camera and lower detected biomass detected per trap night (Figure 6).

## 4 DISCUSSION

### 4.1 Mammal diversity and species richness

Our study represents one of the most comprehensive field‐based investigation of the relationship between elevational change and large (>500 g) mammal communities to date. We found that species richness and Shannon diversity declined consistently as elevation increased and gross primary productivity decreased with the highest species richness was found in the Montane Forest zone and the lowest in the Afro‐Alpine and Rock habitats which is consistent with Chao species estimates we calculated (Figure S5). Thus, our results support the energy richness hypothesis (Wright, 1983) but not the mid‐domain effect (Colwell & Lees, 2000) for larger mammals.

Anthropogenic changes strongly affect mammal communities. We found that a low relative abundance of native mammals in the lowest elevation, which could be related to the negative impacts of human disturbance. Indeed, on a global scale, human impact is much larger in the lowlands (Nogués‐Bravo et al., 2008). However, the risks of human disturbance on montane habitats continues to climb elevation worldwide resulting in encroachment zones that threaten to engulf border zones of protected spaces similar to Mt Kenya's Montane Forest (Feng et al., 2023). The lowest elevation Agro‐Forestry plantations had the second highest species richness among vegetation zones, but showed a low relative abundance of native mammals, presumably due to competition with livestock and hunting. In the absence of the habitat fragmentation and land conversion of the Agro‐Forestry zone, we would expect similar, if not greater, levels of wildlife species richness and relative abundance with decreasing elevation and increasing GPP, a pattern found not just in mammals but in other clades like herpetofauna (Malonza, 2015). Due to the wide range of climatic zones surrounding mountains, our results may differ from patterns on other slopes of Mt Kenya. Specifically, the northern slope features a wider Ericaceous belt but lacks a bamboo or much of a Montane Forest belt due to much drier climate conditions (Allen, 1991; Young, 1984), and the Montane Forest proceeds to much lower elevations on the wetter south‐eastern slope.

### 4.2 Habitat, mid‐elevation trends, and species specialization

The patterns we observed in large mammal species diversity paralleled those in recent research on Africa's highest mountain, Mt Kilimanjaro (Gebert et al., 2019), situated about 400 km south of Mt. Kenya, where large mammal species richness also declined with increasing elevation. However, carnivores on Mt Kilimanjaro were not detected above 3880 m, whereas we detected several carnivore species (leopard, spotted hyena, slender mongoose (*Galerella sanguinea*)) beyond 4500 m. Additional differences in mammal communities across the two mountains may be due to increased human presence along the elevational gradient on Mt Kilimanjaro creating niches for some species that may not have been present without human influence (e.g. crested porcupine at 3650 m on Mt Kilimanjaro, but not Mt Kenya) and potentially excluding sensitive species from other elevations (e.g. African buffalo and bushbuck on Mt Kenya, but not Mt Kilimanjaro). On both mountains, large mammal species richness declined as elevation increased and productivity decreased between 2400 and 4700 m, providing future support for the energy‐richness hypothesis (Srivastava et al., 1998). Furthermore, the close parallel between relative abundance and productivity does, from our work, lend credence to the many individuals hypothesis (Wright, 1983).

This pattern of declining diversity with increasing elevation contrasts with results from many other studies that found a mid‐domain (middle‐elevation peak) effect (Herzog et al., 2005; Hu et al., 2022; McCain, 2005). Small mammal surveys conducted on different parts of Mt Kenya (Musila et al., 2019; Onditi et al., 2022) showed that the pattern may vary depending on which side of the mountain was surveyed. Our results may deviate from the mid‐domain effect, in part, because larger mammals may not be able to specialize on narrow bands of habitat due to their larger space requirements and lower densities compared to small mammals (Beasley & Rhodes, 2010). Due to their smaller home ranges, small mammals may be more likely to be found in single habitat type resulting in high species richness in a relatively small geographic area (Rickart et al., 2011; Schlinkert et al., 2016) which may partially explain why contemporaneous small mammal studies on other parts of the mountain found some evidence for mid‐domain effects in non‐volant small mammal species richness (Musila et al., 2019; Onditi et al., 2022). Second, it is possible that large mammal diversity does show a mid‐domain effect, but that it is spread over a larger elevational gradient. Indeed, the Mt Kilimanjaro study, which focused on elevational ranges from 750 to 4500 m, did find a mid‐domain effect but that the peak diversity was around 2100 m, roughly 200 m lower in elevation than our lowest camera trap (Gebert et al., 2019), a pattern that was similarly observed in a similar elevation study in the Himalayas (Hu et al., 2022). A study conducted in the Laikipia region, immediately west of Mt Kenya, observed an average large mammal species richness of 48 species in protected landscapes between 1600 and 2100 m elevation (Kinnaird & O'Brien, 2012). However, the reality that many regions surrounding the lower slopes of mountains are severely impacted by human activity makes it extremely challenging to disentangle mid‐domain effects from the effects of human disturbance.

### 4.3 Community composition change between habitat types

We observed substantial differences in animal communities in unprotected habitats (Agro‐Forestry) versus adjacent protected Montane Forest habitats administered by the Kenya Wildlife Service. There are several reasons why wildlife detections were limited in the Agro‐Forestry zone. Firstly, wild herbivores venturing out of the Montane Forest and into the Agro‐Forestry zone likely face a high degree of resource competition with domestic livestock, supported by our finding that raw livestock detections outnumbered wildlife detections by a margin of four to one. Additionally, many wildlife species are illegally hunted because they are considered pests (Graham & Ochieng, 2008) or as bushmeat to supplement local farmers' diets (Menz, 2014). The beta‐diversity analyses showed that the apparently high species diversity of the Agro‐Forestry area is deceptive because the mammal community includes severely reduced abundance of native mammals and large numbers of domestic species. Some species like common duiker are likely to be found in the Agro‐Forestry belt as a result of the more open habitat created through land clearance and absence of the prime Montane Forest that has historically been at that elevation. Within the park common duiker were absent from the forested region adjacent to the Agro‐Forestry zone, but were frequently detected in the high elevation open habitat of the Ericaceous zone. Another open habitat species also found in the Agro‐Forestry zone, the steenbok, whose solitary nature might make them more adept at tolerating human and domestic animal co‐habitation, is noted for having a habitat preference for more arid environments (Du Toit, 1993; Leakey et al., 1999).

**FIGURE 7:**
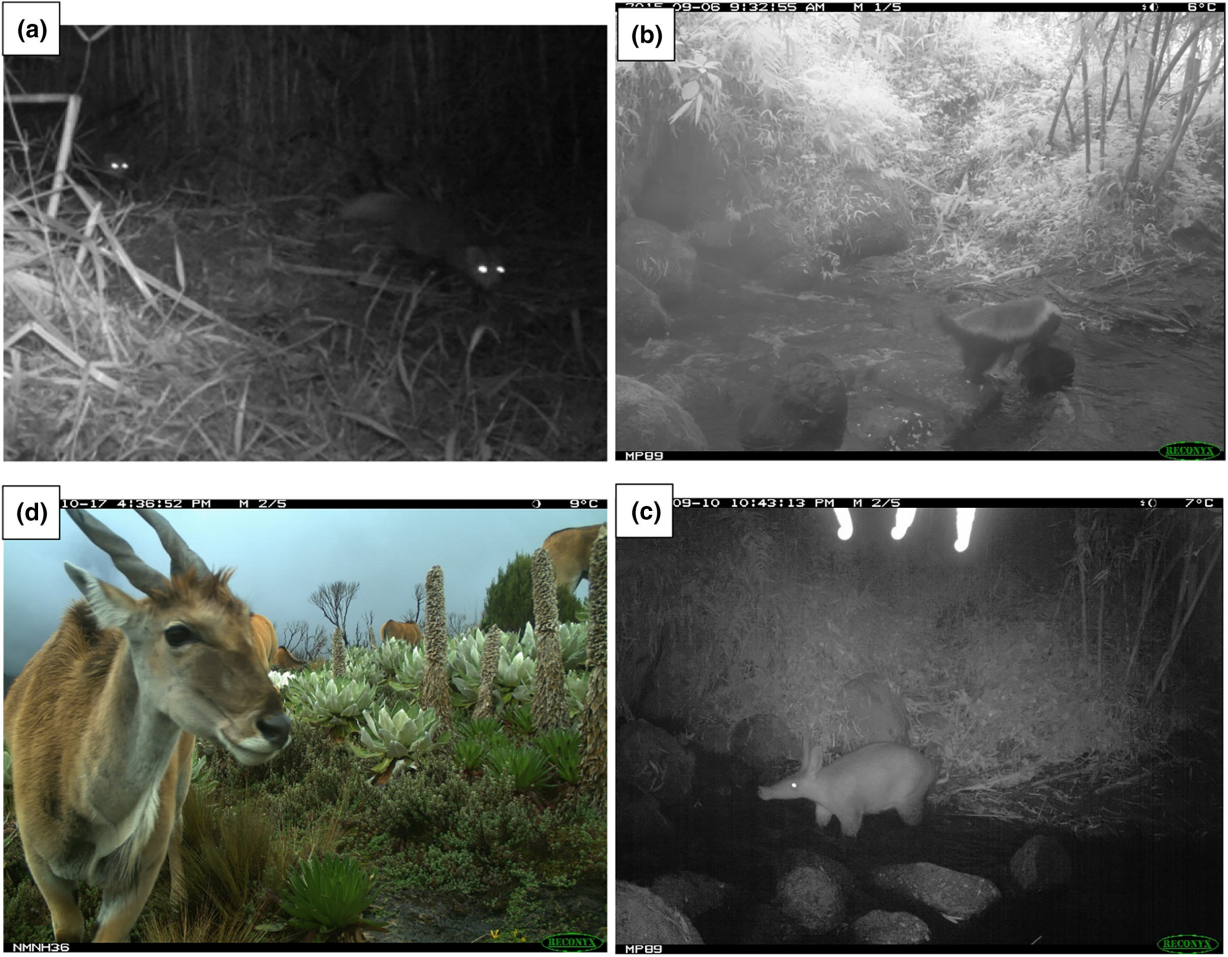
Selected captures of notable mammals. Clockwise from top left: (a) a pair of clandestine Jackson's mongoose, having not been scientifically documented on Mt Kenya since 1923, using a bamboo stand; (b) a honey badger and (c) an aardvark both using water features on Mt Kenya, where there had been no previous records of them ranging; (d) a group of eland at high elevation using the Afro‐Alpine landscape.

Aside from revealing broad elevation patterns in mammal species richness, our study also revealed exciting new records of certain species on the mountain (Figure 7; Table S1). First, we documented Jackson's mongoose (*Bdeogale jacksoni*), which had become so rare it disappeared from comprehensive Mt Kenya mammal lists more than three decades ago (Young & Evans, 1993). We observed Jackson's mongoose on three occasions within a narrow elevational band (2500–2700 m), thus suggesting a previously undocumented surviving population of this IUCN listed “Near Threatened” species (De Luca et al., 2015). Second, we detected two widespread species known mostly from lower‐elevation drylands: honey badger (*Mellivora capensis*) and aardvarks (*Orycteropus afer*), recorded for the first time on Mt Kenya from this study. Eland were the only large herbivore our cameras detected at elevations higher than 4000 m, the lone survivor of a high mountain mammal community that, as recently as 50 years ago, included elephant (*Loxodonta africana*), plains zebra (*Equus quagga*), hartebeest (*Alcelaphus buselaphus* **)**, and African buffalo (*Syncerus caffer*) (Young & Evans, 1993). Our highest detections of elephants were in the Montane Forest and our highest detections of buffalo were in the Ericaceous zone, hundreds of meters lower in elevation than these species have been recorded previously. The low number of elephant detections matches speculation within local conservation organizations that elephants numbers across the Mt Kenya habitat region may be decreasing as a result of major historical movement corridors (Meinertzhagen, 1957) being closed due to land use conversion resulting in an a limited ability to reach the mountain from other protected wildlife areas (Nyaligu & Weeks, 2013).

There were also five species that were notably absent in our survey. Black rhino (*Diceros bicornis*) and African lion (*Panthera leo*) have been extirpated from Mt Kenya with the last published sightings in 1983 and 1977 respectively (Young & Evans, 1993). We did not record endangered African wild dogs (*Lycaon pictus*), several packs of which were observed as high as 3900 m on the mountain as recently as 1967 (Coe, 1967). Mountain bongo (*Tragelaphus eurycerus isaaci*) still survive in the National Park after being nearly hunted to extinction, and are a species of particular interest given ongoing efforts through KWS and private party partnerships to revive the population on Mt Kenya (Kenya Wildlife Service, 2010). Our camera traps transected ideal habitat (Estes et al., 2008) for all of these species on Mt Kenya but we had zero detections. However, we recognize that year‐round sampling could reveal different patterns for species like mountain bongo that are known to still exist on some parts of Mt Kenya.

Our data represent the most exhaustive record of large mammal biodiversity on Mt Kenya conducted since at least the first Roosevelt Expedition over a century ago. From a practical conservation perspective, the changes in community relatedness as it pertains to habitat, where some species exist in large numbers in only one or two habitats and relatively low numbers in the rest, suggests that the whole range of natural habitats must be protected to maintain the total species richness. This concern is particularly relevant for Mt Kenya, given the combination of both low latitudes and large elevational range (Tenorio et al., 2023). It is also worth noting that the area of highest species richness abuts the human modified landscape and is most at risk of habitat fragmentation and land use conversion, a combination that has been projected to be troubling for similar montane biodiversity hotspots, which also face pressures of anthropogenic localized disturbance (Feng et al., 2023) as well as climate change (Iturralde‐Pólit et al., 2017; Ye et al., 2018). Given the expected future climate change in the region (Konecky et al., 2014; Notter et al., 2007; Waititu et al., 2022), we also think this topic will become even more important for evaluating the importance of elevational gradients in protecting biodiversity. These findings reveal the unique nature of this landscape and emphasizes the need for governmental and NGO conservation organizations to continue their essential work to protecting the mammal communities that exist in these rare Afromontane and Afroalpine habitat systems.

## AUTHOR CONTRIBUTIONS

**Matthew H. Snider:** Data curation (supporting); formal analysis (lead); visualization (lead); writing – original draft (lead). **Kristofer M. Helgen:** Conceptualization (equal); funding acquisition (equal); methodology (equal); project administration (equal); writing – review and editing (equal). **Hillary S. Young:** Conceptualization (equal); funding acquisition (equal); methodology (equal); resources (equal); writing – review and editing (equal). **Bernard Agwanda:** Conceptualization (equal); investigation (equal); methodology (equal); project administration (equal); resources (equal); writing – review and editing (equal). **Stephanie Schuttler:** Data curation (equal); investigation (equal); methodology (equal). **Georgia C. Titcomb:** Data curation (equal); formal analysis (equal); investigation (equal); validation (equal); writing – review and editing (equal). **Douglas Branch:** Data curation (equal); investigation (equal); methodology (equal); writing – review and editing (equal). **René Dommain:** Conceptualization (equal); data curation (equal); investigation (equal); methodology (equal); validation (equal); writing – review and editing (equal). **Roland Kays:** Conceptualization (equal); data curation (equal); formal analysis (equal); funding acquisition (equal); investigation (equal); methodology (equal); project administration (equal); resources (equal); supervision (equal); visualization (supporting); writing – review and editing (equal).

## CONFLICT OF INTEREST STATEMENT

No conflicts of interest are known between any co‐author and outside entities regarding fiscal, board membership, consultancy, or any other overlap.

## Supporting information


Appendix S1

## Data Availability

The data and images from this study are available at Wildlife Insights: Kays et al. (2015).

## References

[ece311151-bib-0001] Agnew, A. (2013). Upland Kenya wild flowers and ferns: A Flora of the flowers, ferns, grasses, and sedges of Highland Kenya. East Africa Natural History Society.

[ece311151-bib-0002] Allen, I. A. (1991). The flora, fauna, and ecology of Mount Kenya and Kilimanjaro. In I. A. Allen (Ed.), Guild to Mount Kenya and Kilimanjaro (pp. 37–49). Mountain Club of Kenya.

[ece311151-bib-0003] Arnold, T. W. (2010). Uninformative parameters and model selection using Akaike's information criterion. The Journal of Wildlife Management, 74(6), 1175–1178. 10.1111/j.1937-2817.2010.tb01236.x

[ece311151-bib-0004] Baker, B. H. (1967). Geology of the Mount Kenya area (p. 78). Ministry of Environment and Natural Resources, Mines and Geological Department, Report No. 79.

[ece311151-bib-0005] Baselga, A. , & Orme, C. D. L. (2012). Betapart: An R package for the study of beta diversity. Methods in Ecology and Evolution, 3, 808–812. 10.1111/j.2041-210X.2012.00224.x

[ece311151-bib-0006] Beasley, J. C. , & Rhodes, O. E. (2010). Influence of patch‐ and landscape‐level attributes on the movement behavior of raccoons in agriculturally fragmented landscapes. Canadian Journal of Zoology, 88, 161–169. 10.1139/Z09-137

[ece311151-bib-0007] Beentje, H. (1994). Kenya trees, shrubs, and lianas. National Museums of Kenya.

[ece311151-bib-0008] Bussman, R. W. (1994). *The forests of Mount Kenya (Kenya): vegetation, ecology, destruction and management of a tropical mountain forest ecosystem* [University of Bayreuth]. https://www.academia.edu/15418469/the_forests_of_mount_kenya_kenya_‐_vegetation_ecology_destruction_and_management_of_a_tropical_mountain_forest_ecosystem

[ece311151-bib-0009] Butynski, T. , & De Jong, Y. A. (2017). Biogeography, taxonomy, abundance, and conservation status of the primates of northeast Uganda and West Kenya (pp. 39–40).

[ece311151-bib-0010] Coe, M. J. (1967). The ecology of the alpine zone of Mount Kenya. Monographiae Biologicae, 17, 136.

[ece311151-bib-0011] Colwell, R. , & Lees, D. (2000). The mid‐domain effect: Geometric constraints on the geography of species richness. Trees, 15(2), 70–76. 10.1086/382056 10652559

[ece311151-bib-0012] De Luca, W. , Rovero, F. , & Do Linh San, E. (2015). Bdeogale jacksoni. The IUCN Red List of Threatened Species: Vol. e.T2675A45 . 10.2305/IUCN.UK.2008.RLTS.T2675A9466797.en

[ece311151-bib-0013] Dirnböck, T. , Essl, F. , & Rabitsch, W. (2011). Disproportional risk for habitat loss of high‐altitude endemic species under climate change. Global Change Biology, 17(2), 990–996. 10.1111/j.1365-2486.2010.02266.x

[ece311151-bib-0014] Dommain, R. , Riedl, S. , Olaka, L. A. , Demenocal, P. , Deino, A. L. , Bernhart Owen, R. , Muiruri, V. , Müller, J. , Potts, R. , Strecker, A. , Edited, M. R. , & Rinaldo, A. (2022). Holocene bidirectional river system along the Kenya rift and its influence on East African faunal exchange and diversity gradients. Proceedings of the National Academy of Sciences of the United States of America, 119(28), 1–11. 10.1073/pnas.2121388119 PMC9282390 35759654

[ece311151-bib-0015] Downing, T. A. , Olago, D. O. , Nyumba, T. O. , Peacock, M. M. , & Young, T. P. (2023). Varied plant species' responses to climate and environmental change on Mount Kenya after 40 years. African Journal of Ecology, 62, e13207. 10.1111/aje.13207

[ece311151-bib-0016] Du Toit, J. (1993). The feeding ecology of a very small ruminant, the steenbok. African Journal of Ecology, 31, 35–48.

[ece311151-bib-0017] Eckert, S. , Kiteme, B. , Njuguna, E. , & Zaehringer, J. G. (2017). Agricultural expansion and intensification in the foothills of Mount Kenya: A landscape perspective. Remote Sensing, 9(8), 1–20. 10.3390/rs9080784

[ece311151-bib-0018] Estes, L. D. , Okin, G. S. , Mwangi, A. G. , & Shugart, H. H. (2008). Habitat selection by a rare forest antelope: A multi‐scale approach combining field data and imagery from three sensors. Remote Sensing of Environment, 112, 2033–2050. 10.1016/j.rse.2008.01.004

[ece311151-bib-0019] Feng, L. , Ma, X. , Hughes, A. C. , & Feng, G. (2023). Elevation range and contemporary climate determine the taxonomic, functional and phylogenetic diversity of forest mammals. Biodiversity and Conservation, 32(14), 4651–4664. 10.1007/s10531-023-02715-7

[ece311151-bib-0020] Gebert, F. , Njovu, H. K. , Treydte, A. C. , Steffan‐Dewenter, I. , & Peters, M. K. (2019). Primary productivity and habitat protection predict elevational species richness and community biomass of large mammals on Mt. Kilimanjaro. Journal of Animal Ecology, 88(12), 1860–1872. 10.1111/1365-2656.13074 31410849

[ece311151-bib-0021] Graham, M. D. , & Ochieng, T. (2008). Uptake and performance of farm‐based measures for reducing crop raiding by elephants *Loxodonta africana* among smallholder farms in Laikipia District, Kenya. Oryx, 42(1), 76–82. 10.1017/S0030605308000677

[ece311151-bib-0022] Hartig, F. (2020). *DHARMa: residual diagnostics for hierarchical (multi‐level/mixed) regression models* (R package version 0.3). https://cran.r‐project.org/web/packages/DHARMa/vignettes/DHARMa.html

[ece311151-bib-0023] Hedberg, O. (1951). Vegetation belts of the east African Mountains: Results of the Swedish East Africa expedition 1948. Almqvist & Wiksells Boktryckeri.

[ece311151-bib-0024] Herzog, S. K. , Kessler, M. , & Bach, K. (2005). The elevational gradient in Andean bird species richness at the local scale: A foothill peak and a high‐elevation plateau. Ecography, 28, 209–222.

[ece311151-bib-0025] Hu, Y. , Gibson, L. , Hu, H. , Ding, Z. , Zhou, Z. , Li, W. , Jiang, Z. , & Scheffers, B. R. (2022). Precipitation drives species accumulation whereas temperature drives species decline in Himalayan vertebrates. Journal of Biogeography, 49(12), 2218–2230. 10.1111/jbi.14499

[ece311151-bib-0026] Iturralde‐Pólit, P. , Dangles, O. , Burneo, S. F. , & Meynard, C. N. (2017). The effects of climate change on a mega‐diverse country: Predicted shifts in mammalian species richness and turnover in continental Ecuador. Biotropica, 49(6), 821–831. 10.1111/btp.12467

[ece311151-bib-0027] Kays, R. , Arbogast, B. S. , Baker‐Whatton, M. , Beirne, C. , Boone, H. M. , Bowler, M. , Burneo, S. F. , Cove, M. V. , Ding, P. , Espinosa, S. , Gonçalves, A. L. S. , Hansen, C. P. , Jansen, P. A. , Kolowski, J. M. , Knowles, T. W. , Lima, M. G. M. , Millspaugh, J. , McShea, W. J. , Pacifici, K. , … Spironello, W. R. (2020). An empirical evaluation of camera trap study design: How many, how long and when? Methods in Ecology and Evolution, 11(6), 700–713. 10.1111/2041-210X.13370

[ece311151-bib-0028] Kays, R. , Helgen, K. , Young, H. S. , Agwanda, B. , Schuttler, S. , Titcomb, G. C. , Branch, D. , & Snider, M. H. (2015). Mount Kenya Survey . http://n2t.net/ark:/63614/w12006138. wildlifeinsights.org 10.1002/ece3.11151 PMC11004549 38601855

[ece311151-bib-0029] Kays, R. , Hody, A. , Jachowski, D. , & Parsons, A. W. (2021). Empirical evaluation of the spatial scale and detection process of camera trap surveys. Movement Ecology, 9(41), 1–13. 34391486 10.1186/s40462-021-00277-3 PMC8364038

[ece311151-bib-0030] Kays, R. , Tilak, S. , Kranstauber, B. , Jansen, P. A. , Carbone, C. , Rowcliffe, M. J. , Fountain, T. , Eggert, J. , & He, Z. (2010). Monitoring wild animal communities with arrays of motion sensitive camera traps. *ArXiv*. 1–22. http://arxiv.org/abs/1009.5718

[ece311151-bib-0031] Kehlenbeck, K. , Kindt, R. , Sinclair, F. L. , Simons, A. J. , & Jamnadass, R. (2011). Exotic tree species displace indigenous ones on farms at intermediate altitudes around Mount Kenya. Agroforestry Systems, 83, 133–147. 10.1007/s10457-011-9413-4

[ece311151-bib-0032] Kenya Wildlife Service . (2010). Mt Kenya Ecosystem Management Plan, 2010–2020. MKE Management Plan (2010–2020) (pp. 53–55). Kenya Wildlife Service, Ministry of Tourism and Wildlife.

[ece311151-bib-0033] Kingdon, J. , Happold, D. , Hoffmann, M. , Butynski, T. , Happold, M. , & Kalina, J. (2013). Mammals of Africa. Bloomsbury Publishing.

[ece311151-bib-0034] Kinnaird, M. F. , & O'Brien, T. G. (2012). Effects of private‐land use, livestock management, and human tolerance on diversity, distribution, and abundance of large African mammals. Conservation Biology, 26(6), 1026–1039. 10.1111/j.1523-1739.2012.01942.x 23082891

[ece311151-bib-0035] Konecky, B. , Russell, J. , Huang, Y. , Vuille, M. , Cohen, L. , & Street‐Perrott, F. A. (2014). Impact of monsoons, temperature, and CO_2_ on the rainfall and ecosystems of Mt. Kenya during the common era. Palaeogeography, Palaeoclimatology, Palaeoecology, 396, 17–25. 10.1016/j.palaeo.2013.12.037

[ece311151-bib-0036] Leakey, L. N. , Milledge, S. A. H. , Leakey, S. M. , & Edung, J. (1999). Diet of striped hyaena in northern Kenya. African Journal of Ecology, 37, 314–326.

[ece311151-bib-0037] Malhi, Y. , Girardin, C. A. J. , Goldsmith, G. R. , Doughty, C. E. , Salinas, N. , Metcalfe, D. B. , Huaraca Huasco, W. , Silva‐Espejo, J. E. , del Aguilla‐Pasquell, J. , Farfán Amézquita, F. , Aragão, L. E. O. C. , Guerrieri, R. , Ishida, F. Y. , Bahar, N. H. A. , Farfan‐Rios, W. , Phillips, O. L. , Meir, P. , & Silman, M. (2017). The variation of productivity and its allocation along a tropical elevation gradient: A whole carbon budget perspective. New Phytologist, 214, 1019–1032. 10.1111/nph.14189 27768811

[ece311151-bib-0038] Malonza, P. K. (2015). Patterns of reptile and amphibian species richness along elevational gradients in Mt. Kenya. Zoological Research, 36(6), 342–347. 10.13918/j.issn.2095-8137.2015.6.342 26646571 PMC4771954

[ece311151-bib-0039] Marris, E. (2007). The escalator effect. Nature Climate Change, 1(712), 94–96. 10.1038/climate.2007.70

[ece311151-bib-0040] McCain, C. M. (2004). The mid‐domain effect applied to elevational gradients: Species richness of small mammals in Costa Rica. Journal of Biogeography, 31, 19–31.

[ece311151-bib-0041] McCain, C. M. (2005). Elevational gradients in diversity of small mammals. Ecology, 86(2), 366–372. 10.1890/03-3147

[ece311151-bib-0042] McCain, C. M. , & Colwell, R. K. (2011). Assessing the threat to montane biodiversity from discordant shifts in temperature and precipitation in a changing climate. Ecology Letters, 14(12), 1236–1245. 10.1111/j.1461-0248.2011.01695.x 21981631

[ece311151-bib-0043] McShea, W. J. , Forrester, T. , Costello, R. , He, Z. , & Kays, R. (2016). Volunteer‐run cameras as distributed sensors for macrosystem mammal research. Landscape Ecology, 31, 55–66. 10.1007/s10980-015-0262-9

[ece311151-bib-0044] Meinertzhagen, R. (1957). Kenya diary, 1902–1906. Oliver and Boyd.

[ece311151-bib-0045] Menz, A. (2014). A means to alleviate the bushmeat crisis? The feasibility of establishing sustainable grasscutter farms in Kenya. Consilience: The Journal of Sustainable Development, 13(1), 130–164.

[ece311151-bib-0046] Mittermeier, R. A. , Gil, P. R. , Pilgrim, J. D. , Brooks, T. M. , Mittermeier, C. G. , Lamoreux, J. , & Da Fonseca, G. A. B. (2004). Hotspots revisited: Earth’s biologically richest and most endangered terrestrial ecoregions. Cemex. Cemex Books on Nature.

[ece311151-bib-0082] Mittermeier, R. A. , Turner, W. R. , Larsen, F. W. , Brooks, T. M. , & Gascon, C. (2011). Global biodiversity conservation: The critical role of hotspots. In F. Zachos , & J. Habel (Eds.), Biodiversity Hotspots (pp. 3–22). Springer. 10.1007/978-3-642-20992-5_1

[ece311151-bib-0047] Musila, S. , Chen, Z.‐Z. , Li, Q. , Yego, R. , Zhang, B. , Onditi, K. , Muthoni, I. , He, S.‐W. , Omondi, S. , Mathenge, J. , Kioko, E. N. , & Jiang, X.‐L. (2019). Diversity and distribution patterns of non‐volant small mammals along different elevation gradients on Mt. Kenya, Kenya. Zoological Research, 40(1), 53–60. 10.24272/j.issn.2095-8137.2019.004 30581186 PMC6350105

[ece311151-bib-0048] Nakashima, Y. (2018). Estimating animal density without individual recognition using information derivable exclusively from camera traps. Journal of Applied Ecology, 55, 735–744. 10.1111/1365-2664.13059

[ece311151-bib-0049] Nicholson, S. E. (2017). Climate and climatic variability of rainfall over eastern Africa. Reviews of Geophysics, 55(3), 590–635. 10.1002/2016RG000544

[ece311151-bib-0050] Nogués‐Bravo, D. , Araújo, M. B. , Romdal, T. , & Rahbek, C. (2008). Scale effects and human impact on the elevational species richness gradients. Nature, 453(7192), 216–219. 10.1038/nature06812 18464741

[ece311151-bib-0051] Notter, B. , MacMillan, L. , Viviroli, D. , Weingartner, R. , & Liniger, H. P. (2007). Impacts of environmental change on water resources in the Mt. Kenya region. Journal of Hydrology, 343(3–4), 266–278. 10.1016/j.jhydrol.2007.06.022

[ece311151-bib-0052] Nyaligu, M. O. , & Weeks, S. (2013). An elephant corridor in a fragmented conservation landscape: Preventing the isolation of Mount Kenya National Park and National Reserve. Parks, 19(1), 91–102.

[ece311151-bib-0053] Oksanen, A. J. , Blanchet, F. G. , Kindt, R. , Legen, P. , Minchin, P. R. , Hara, R. B. O. , Simpson, G. L. , Solymos, P. , & Stevens, M. H. H. (2016). Vegan: Community ecology package. In … ecology package . http://mirror.bjtu.edu.cn/cran/web/packages/vegan/vegan.pdf

[ece311151-bib-0054] Onditi, K. O. , Song, W. Y. , Li, X. Y. , Chen, Z. Z. , Li, Q. , He, S. W. , Musila, S. , Kioko, E. , & Jiang, X. L. (2022). Patterns and predictors of small mammal phylogenetic and functional diversity in contrasting elevational gradients in Kenya. Frontiers in Ecology and Evolution, 9, 1–18. 10.3389/fevo.2021.742524

[ece311151-bib-0055] Pan, X. , Ding, Z. , Hu, Y. , Liang, J. , Wu, Y. , Si, X. , Guo, M. , Hu, H. , & Jin, K. (2016). Elevational pattern of bird species richness and its causes along a central Himalaya gradient, China. PeerJ, 4, 1–22. 10.7717/peerj.2636 PMC5101612 27833806

[ece311151-bib-0056] Patterson, B. D. , Stotz, D. F. , Solarit, S. , & Fitzpatrick, J. W. (1998). Contrasting patterns of elevational zonation for birds and mammals in the Andes of southeastern Peru. Journal of Biogeography, 25(3), 593–607.

[ece311151-bib-0057] Pauchard, A. , Milbau, A. , Albihn, A. , Alexander, J. , Burgess, T. , Daehler, C. , Englund, G. , Essl, F. , Evengård, B. , Greenwood, G. B. , Haider, S. , Lenoir, J. , McDougall, K. , Muths, E. , Nuñez, M. A. , Olofsson, J. , Pellissier, L. , Rabitsch, W. , Rew, L. J. , … Kueffer, C. (2016). Non‐native and native organisms moving into high elevation and high latitude ecosystems in an era of climate change: New challenges for ecology and conservation. Biological Invasions, 18, 345–353. 10.1007/s10530-015-1025-x

[ece311151-bib-0058] QGIS Development Team . (2018). QGIS geographic information system. Open Source Geospatial Foundation Project. http://qgis.osgeo.org

[ece311151-bib-0059] Radeloff, V. C. , Dubinin, M. , Coops, N. C. , Allen, A. M. , Brooks, T. M. , Clayton, M. K. , Costa, G. C. , Graham, C. H. , Helmers, D. P. , Ives, A. R. , Kolesov, D. , Pidgeon, A. M. , Rapacciuolo, G. , Razenkova, E. , Suttidate, N. , Young, B. E. , Zhu, L. , & Hobi, M. L. (2019). The dynamic habitat indices (DHIs) from MODIS and global biodiversity. Remote Sensing of Environment, 222, 204–214. 10.1016/j.rse.2018.12.009

[ece311151-bib-0060] Rahbek, C. (1995). The elevational gradient of species richness: A uniform pattern? Ecography, 18(2), 200–205.

[ece311151-bib-0061] Rahbek, C. , Borregaard, M. K. , Antonelli, A. , Colwell, R. K. , Holt, B. G. , Nogues‐bravo, D. , Rasmussen, C. M. Ø. , Richardson, K. , Rosing, M. T. , Whittaker, R. J. , & Fjeldså, J. (2019). Building mountain biodiversity: Geological and evolutionary processes. Science, 365, 1114–1119. 31515384 10.1126/science.aax0151

[ece311151-bib-0062] Rahbek, C. , Borregaard, M. K. , Colwell, R. K. , Dalsgaard, B. , Holt, B. G. , Morueta‐holme, N. , Nogues‐bravo, D. , Whittaker, R. J. , & Fjeldså, J. (2019). Humboldt's enigma: What causes global patterns of mountain biodiversity? Science, 365, 1108–1113. 31515383 10.1126/science.aax0149

[ece311151-bib-0063] Rickart, E. A. , Heaney, L. R. , Balete, D. S. , & Tabaranza, B. R. (2011). Small mammal diversity along an elevational gradient in northern Luzon, Philippines. Mammalian Biology, 76, 12–21. 10.1016/j.mambio.2010.01.006

[ece311151-bib-0064] Roosevelt, T. (1910). P. H. Capstick (Ed.), African game trails: An account of the African wanderings of an American hunter‐naturalist (1st ed.). Charles Scribner's Sons.

[ece311151-bib-0065] Rowcliffe, J. M. , Carbone, C. , Jansen, P. A. , Kays, R. , & Kranstauber, B. (2011). Quantifying the sensitivity of camera traps: An adapted distance sampling approach. Methods in Ecology and Evolution, 2(5), 464–476. 10.1111/j.2041-210X.2011.00094.x

[ece311151-bib-0066] Rowcliffe, J. M. , Field, J. , Turvey, S. T. , & Carbone, C. (2008). Estimating animal density using camera traps without the need for individual recognition. Journal of Applied Ecology, 45(4), 1228–1236. 10.1111/j.1365-2664.2008.01473.x

[ece311151-bib-0067] Sanchez‐Cordero, V. (2001). Elevation gradients of diversity for rodents and bats in Oaxaca, Mexico. Global Ecology and Biogeography, 10, 63–76.

[ece311151-bib-0068] Schlinkert, A. , Schlinkert, H. , Ludwig, M. , Batáry, P. , Holzschuh, A. , Kovács‐hostyánszki, A. , Tscharntke, T. , & Fischer, C. (2016). Forest specialist and generalist small mammals in forest edges and hedges. Wildlife Biology, 22(3), 86–94. 10.2981/wlb.00176

[ece311151-bib-0069] Sekercioglu, C. H. , Schneider, S. H. , Fay, J. P. , & Loarie, S. R. (2008). Climate change, elevational range shifts, and bird extinctions. Conservation Biology, 22(1), 140–150. 10.1111/j.1523-1739.2007.00852.x 18254859

[ece311151-bib-0070] Smith, A. P. , & Young, T. P. (1987). Tropical alpine plant ecology. Annual Review of Ecology and Systematics, 18(1), 137–158. 10.1146/annurev.es.18.110187.001033

[ece311151-bib-0071] Srivastava, D. S. , Lawton, J. H. , Srivastava, D. S. , & Lawton, J. H. (1998). Why more productive sites have more species: An experimental test of theory using tree‐hole communities. The American Naturalist, 152(4), 510–529. 10.1086/286187 18811361

[ece311151-bib-0072] Storch, D. , Bohdalkova, E. , & Okie, J. (2018). The more‐individuals hypothesis revisited: The role of community abundance in species richness regulation and the productivity – Diversity relationship. Ecology Letters, 21, 920–937. 10.1111/ele.12941 29659144

[ece311151-bib-0073] Tattersfield, P. , Warui, C. M. , Seddon, M. B. , & Kiringe, J. W. (2001). Land‐snail faunas of afromontane forests of Mount Kenya, Kenya: Ecology, diversity and distribution patterns. Journal of Biogeography, 28, 841–861.

[ece311151-bib-0074] Tenorio, E. A. , Montoya, P. , Norden, N. , Rodríguez‐Buriticá, S. , Salgado‐Negret, B. , & Gonzalez, M. A. (2023). Mountains exhibit a stronger latitudinal diversity gradient than lowland regions. Journal of Biogeography, 50(6), 1026–1036. 10.1111/jbi.14597

[ece311151-bib-0075] University of Michigan Museum of Zoology . (2020). Animal diversity web . https://animaldiversity.org/

[ece311151-bib-0076] Veldkamp, A. , Schoorl, J. M. , Wijbrans, J. R. , & Claessens, L. (2012). Mount Kenya volcanic activity and the late Cenozoic landscape reorganisation in the upper tana fluvial system. Geomorphology, 145–146, 19–31. 10.1016/j.geomorph.2011.10.026

[ece311151-bib-0077] Waititu, J. M. , Mundia, C. N. , & Sichangi, A. W. (2022). Assessing distribution changes of selected native and alien invasive plant species under changing climatic conditions in Nyeri County, Kenya. PLoS One, 17, 1–23. 10.1371/journal.pone.0275360 PMC9529121 36190975

[ece311151-bib-0078] Wright, D. H. (1983). Species‐energy theory: An extension of species‐area theory. Oikos, 41(3), 496–506.

[ece311151-bib-0079] Ye, X. , Yu, X. , Yu, C. , Tayibazhaer, A. , Xu, F. , Skidmore, A. K. , & Wang, T. (2018). Impacts of future climate and land cover changes on threatened mammals in the semi‐arid Chinese Altai Mountains. Science of the Total Environment, 612(620), 775–787. 10.1016/j.scitotenv.2017.08.191 28866405

[ece311151-bib-0080] Young, T. P. (1984). Kenya's alpine and high forest ecosystems. In V. C. Gilbert (Ed.), Endangered resources for development (pp. 119–137). National Environment and Human Settlements Secretariet.

[ece311151-bib-0081] Young, T. P. , & Evans, M. R. (1993). Alpine vertebrates of Mount Kenya, with particular notes on the rock hyrax. Journal of East African Natural History, 82(202), 55–79.

