# Supplementary material for: Shifting mammal communities and declining species richness along an elevational gradient on Mount Kenya
Source: Ecol Evol. 2024 Apr 9;14(4):e11151. doi: 10.1002/ece3.11151 (PMC11004549; doi:10.1002/ece3.11151)
Supplement: Supplementary file 1 — Appendix S1 [file ECE3-14-e11151-s001.docx]

**Table S1:** Species captured and detection rates across all cameras

| **Common Name** | **Scientific Name** | **Total Count** | **Detection Rate (captures/trap night)** |
| --- | --- | --- | --- |
| Aardvark | *Orycteropus afer* | 12 | 0.0021 |
| African buffalo | *Syncerus caffer* | 83 | 0.0144 |
| African brush-tailed porcupine | *Atherurus africanus* | 1 | 0.0002 |
| African elephant | *Loxodonta africana* | 14 | 0.0024 |
| Black-fronted duiker | *Cephalophus nigrifrons* | 126 | 0.0219 |
| Blue monkey | *Cercopithecus mitis* | 219 | 0.0380 |
| Bohor reedbuck | *Redunca redunca* | 3 | 0.0005 |
| Bushbuck | *Tragelaphus scriptus* | 464 | 0.0806 |
| Bushpig | *Potamochoerus larvatus* | 65 | 0.0113 |
| Common duiker | *Sylvicapra grimmia* | 221 | 0.0384 |
| Common eland | *Taurotragus oryx* | 41 | 0.0071 |
| Crested porcupine | *Hystrix cristata* | 7 | 0.0019 |
| Four-toed hedgehog | *Atelerix albiventris* | 2 | 0.0003 |
| Honey badger | *Mellivora capensis* | 2 | 0.0003 |
| Jackson's mongoose | *Bdeogale jacksoni* | 4 | 0.0007 |
| Leopard | *Panthera pardus* | 113 | 0.0196 |
| Marsh mongoose | *Atilax paludinosus* | 1 | 0.0002 |
| Rock hyrax | *Procavia capensis* | 281 | 0.0488 |
| Serval | *Leptailurus serval* | 32 | 0.0056 |
| Slender mongoose | *Galerella sanguinea* | 11 | 0.0019 |
| Southern tree hyrax | *Dendrohyrax arboreus* | 10 | 0.0017 |
| Spotted hyena | *Crocuta Crocuta* | 245 | 0.0426 |
| Steenbok | *Raphicerus campestris* | 5 | 0.0009 |
| Suni | *Neotragus moschatus* | 763 | 0.1326 |
| Zorilla | *Ictonyx striatus* | 14 | 0.0024 |
| Genets (Common genet, Central African large-spotted genet, unknown genet) | *Genetta maculate* or *Genetta genetta* | 43 | 0.0075 |
| Hares (Cape hare, Scrub hare, unknown hare) | *Lepus saxatilis or Lepus capensis* | 60 | 0.0104 |
| Crested Rat | Lophiomys imhausi, | 2 | 0.0003 |
| Domestic cow | *Bos taurus* | 736 | 0.1279 |
| Domestic dog | *Canis familiaris* | 111 | 0.0193 |
| Domestic goat | *Capra aegagrus hircus* | 141 | 0.0245 |
| Domestic sheep | *Ovis aries* | 1459 | 0.2535 |

**Table S2:** Pairwise comparison in species composition relatedness with the Sørenson Similarity index between different habitat types. Lower numbers indicate those pair habitats are more closely related.

|  | Agro-Forestry | Montane Forest | Bamboo | Ericaceous | Rock |
| --- | --- | --- | --- | --- | --- |
| Montane Forest | 0.45 |  |  |  |  |
| Bamboo | 0.47 | 0.31 |  |  |  |
| Ericaceous | 0.56 | 0.50 | 0.28 |  |  |
| Rock | 0.73 | 0.78 | 0.60 | 0.29 |  |
| Afro-Alpine | 0.64 | 0.78 | 0.60 | 0.29 | 0.33 |


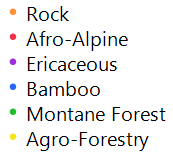


(m)

**Figure S1.** Raw detection rates of native mammals for individual cameras across elevation and habitat zones. There is a clear trend of decreasing detection rate as elevation increases. Due to the topography at near the border between the Agro-Forestry and Montane Forest, a few of the Montane Forest cameras that were on hill slopes were technically lower elevation than a few of the Agro-Forestry cameras deployed on a low rise outside the protected area. The few cameras in the Afro-Alpine and Rock habitat with high detection rates had high activity of rock hyrax (*Procavia capensis*). Domestic animals are not considered in this analysis.


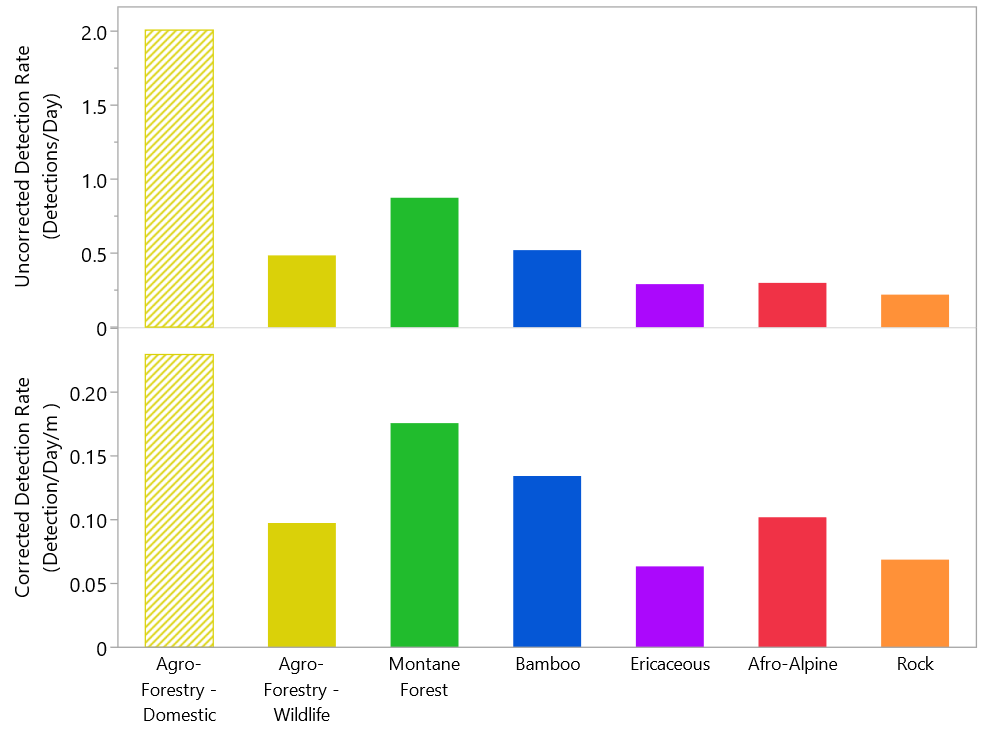


**Figure S2:** Uncorrected and corrected detection rates for domestic and wild animals averaged across each habitat zones. Corrected values used body mass of each detected animal to account for different effective sampling areas per camera. Only two dogs and no livestock were detected in the Montane Forest zone and were excluded from this graph, and no domestic animals were detected in the other habitats within the protected area.


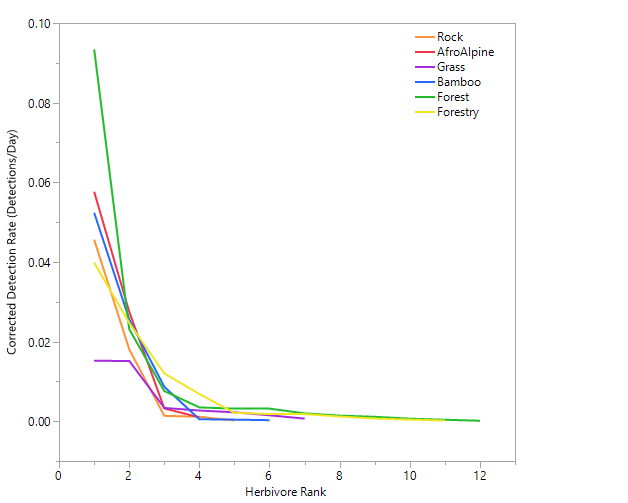

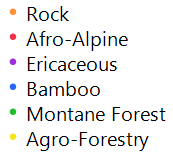

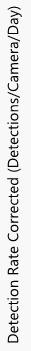
**Figure S3:** Total ranked abundance of herbivores (A) as the x-axis where 1 is the most abundance species, denoted by habitat. Relative abundance measures are adjusted to account for difference in detectability based on body size.

**A**


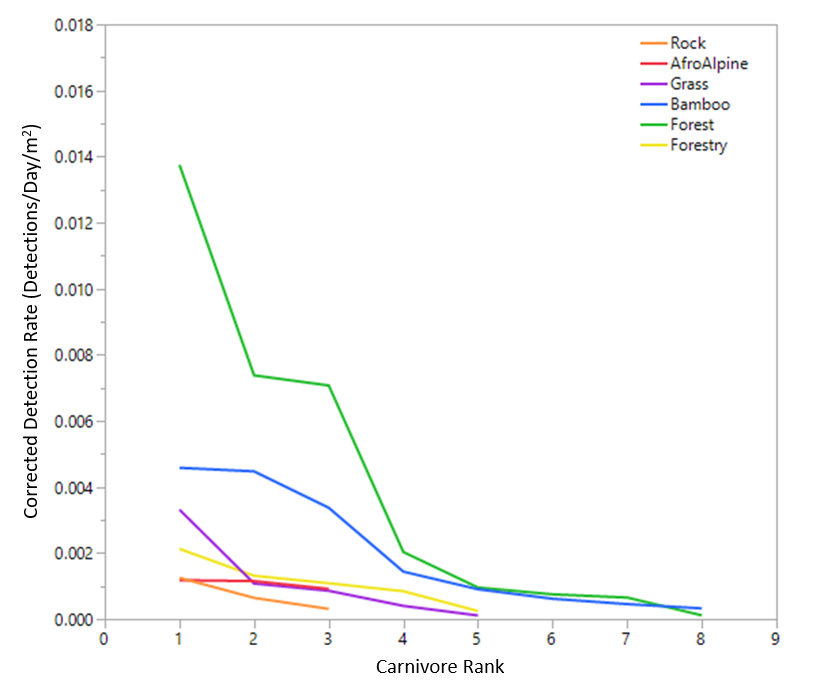

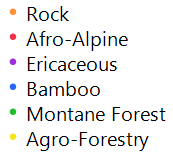

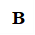


**Figure S4:** Total ranked abundance of carnivores (B) as the x-axis where 1 is the most abundance species, denoted by habitat. Relative abundance measures are adjusted to account for difference in detectability based on body size.


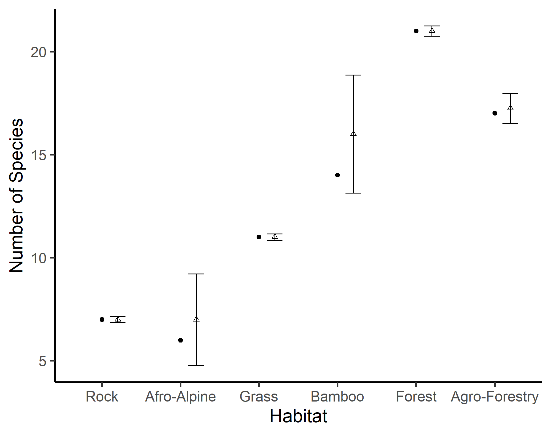


**Figure S5:** As shown in this graph, the estimated number of total species (triangles and standard error bars) for each habitat is very similar to the observed species (points). Bamboo shows the highest potential to have additional undetected species; however, this does not substantially change its rank in terms of species richness for each habitat.


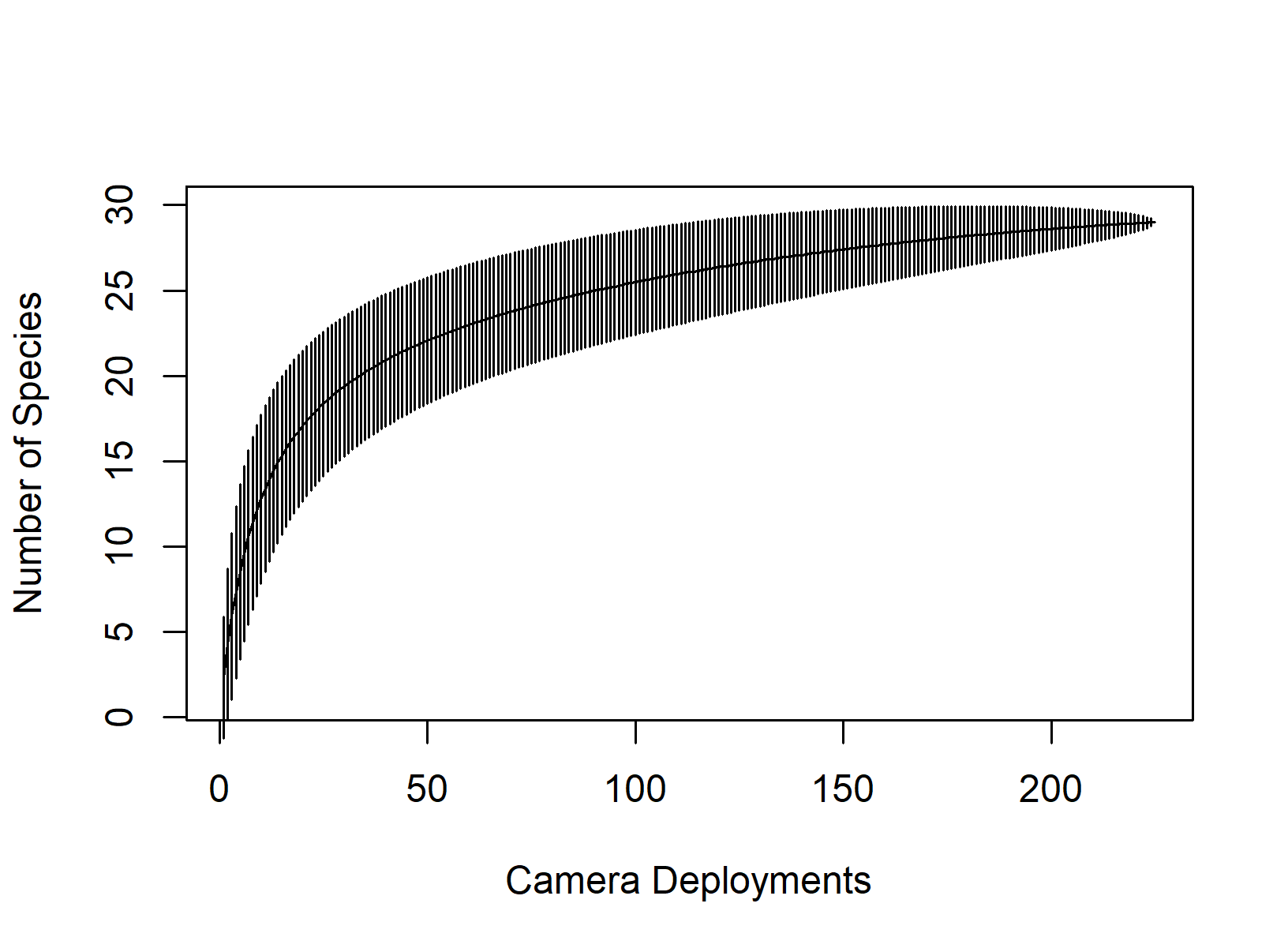


**Figure S5:** As shown by the species accumulation curve, the estimated number of species has levelled-off at approximately 30 over our 219 deployments.
